# News and Miscellany

**Published:** 1904-11

**Authors:** 


### News and Miscellany.

**Every doctor in Texas not already "in it" should at once subscribe for the "Red Back," the live, progressive organ of the Texas profession. Every one in sympathy with the work of the State and county medical societies, and who would know what is doing, should subscribe now.**

Don't fail to read the very excellent paper by Dr. Jordan in this issue.

Dr. R. E. Nicholson and Miss Mary Stone of Brenham are to be married November 24th. Cards received.

Battle & Co., St. Louis, will send free on request, naming "Red Back," twelve illustrations of intestinal parasites; third series.

Dr. J. M. Fry, Wills Point, lost his office and contents by fire October 20th ult. He deplores the loss of his complete file of the "Red Back" (Vol. I, No. 1, July, 1885, to Vol. XX, No. 4, 1904), and the picture of "our Mascot" more than all else, and says he "can't keep shop without the 'Red Back.'"

---

Dr. L. M. Berg, San Antonio, has moved his office from Southern Hotel Drug Store to Moore building, corner Avenue C and Houston Street.

---

Surgeon General Walter Wyman, Marine Hospital Service, was elected President of the Military Surgeon Association at St. Louis, October 15th ult.

---

Dr. Bramford Lewis was elected President of the Mississippi Valley Medical Association at the Cincinnati meeting October 11th (ult.), and Dr. H. E. Tuley, Secretary.

---

Dr. W. R. Blailock, now of Dallas, was elected President of the Tri-State Medical Association (Indian and Oklahoma Territories and Texas) at the Dallas meeting November 5th.

---

Will Repair Your Electric Machines.—Static and all electrical medical apparatus put in running order. I am also agent for electrical and X-ray apparatus. Oliver Brush, 710 Colorado Street, Austin, Texas.

---

$2500 to $3000 unopposed practice to purchaser of residence and thirty acres fine land in country village. Small stock drugs, and telephone line included. Price, $2000. Address Wm. M. Morgan, M. D., McMahan, Texas.

---

Southern Medicine (Savannah, Ga.) and *Gaillard's Medical Journal* (New York City) have consolidated, and appear with the names hyphenated. It is edited and published at Savannah by Dr. W. E. Fitch. Eastern office, 90 William Street, New York.

---

Quarantine Changes.—Dr. J. H. Florence has been appointed quarantine inspector at Brownsville, Texas, vice Dr. A. S. Wolff, deceased, and Dr. J. J. Shaver has been appointed at Aransas Pass (Rockport), to take the place made vacant by the transfer of Dr. Florence.

---

Medical College Consolidation.—The Dallas Medical College has consolidated with the Baylor University College of Medicine, and the new institution will be known in the future as the Dallas College of Physicians and Surgeons, the Medical Department of Baylor University. The combined matriculation will number 250 students. The new consolidation carries with it membership in the Southern Association of Medical Colleges. The majority of the members of each faculty will have places provided in the consolidation.

---

Another Deadly Danger.—Deodorized wood (Methyl) alcohol is being colored and made the basis of whisky and sold as such. Thirty death from this cause occurred in a limited area of New York City recently. Wood alcohol should be classed with *poisons,* and its sale restricted. Soon, patent medicines will be made of it, as it is cheaper than alcohol.

---

A "Baptist Memorial Sanitarium," to cost $200,000, is to be built at Dallas at once, by the Baptist people of Texas. It is to be used by the Medical Department of the Baylor (Baptist) University. Ground was formally broken for the building November 5th inst., with great ceremony and speechmaking. It was during the meeting of the Tri-State Medical Association.

---

New Orleans Polyclinic.—Eighteenth annual session opens November 7, 1904, and closes May 10, 1905. Physicians will find the Polyclinic an excellent means for posting themselves upon modern progress in all branches of medicine and surgery. The specialties are fully taught, including laboratory and cadaveric work. For further information, address New Orleans Polyclinic, postoffice box 797, New Orleans, La.

---

Brag is a good dog; holdfast is better. The Danbury Medical Printing Co., in renewing contract for the fifteenth year, says: "Vass Chemical Co. is doing very little medical journal advertising, but the 'Red Back' has been kept on the list."

Rio Chemical Co. say: "We will further show our appreciation by increasing our space in 1905" (twenty years' continuous patronage). Most of my patrons say the same, and have renewed for the twentieth consecutive year. That testifies to the value of the "Red Back" as an advertising medium.

---

Who's Who in Medicine?—The American Medical Association purposes to print and publish a directory of the physicians belonging to the various State and Territorial Associations and their component branches. While ultimately it is expected to make it a complete directory of all licensed physicians, for the present it will only include those in good standing in the organization. Ready January 1, 1905. Reputable physicians do not like to be classed with quacks, pretenders, patent medicine venders, etc., as is the case at present.

When such directory is published, those who are "not in it" will be considered "nobody in particular," and will be discriminated against in business. Insurance companies always ask of an applicant to be medical examiner: "Is he a member of the State Medical Association."

